# Preferences across the Menstrual Cycle for Masculinity and Symmetry in Photographs of Male Faces and Bodies

**DOI:** 10.1371/journal.pone.0004138

**Published:** 2009-01-07

**Authors:** Marianne Peters, Leigh W. Simmons, Gillian Rhodes

**Affiliations:** 1 Centre for Evolutionary Biology, School of Animal Biology, University of Western Australia, Crawley, Australia; 2 School of Psychology, University of Western Australia, Crawley, Australia; London School of Economics, United Kingdom

## Abstract

**Background:**

Previous studies have shown that women increase their preference for masculinity during the fertile phase of the menstrual cycle. Evidence for a similar preference shift for symmetry is equivocal. These studies have required participants to choose between subtle variations in computer-generated stimuli, and preferences for more natural stimuli have not been investigated.

**Methodology/Principal Findings:**

Our study employed photographs of individual males to investigate women's preferences for face and body masculinity and symmetry across the menstrual cycle. We collected attractiveness ratings from 25 normally cycling women at high- and low-fertility days of the menstrual cycle. Attractiveness ratings made by these women were correlated with independent ratings of masculinity and symmetry provided by different sets of raters. We found no evidence for any cyclic shift in female preferences. Correlations between attractiveness and masculinity, and attractiveness and symmetry did not differ significantly between high- and low-fertility test sessions. Furthermore, there was no significant difference between high- and low-fertility ratings of attractiveness.

**Conclusions:**

These results suggest that a menstrual cycle shift in visual preferences for masculinity and symmetry may be too subtle to influence responses to real faces and bodies, and subsequent mate-choice decisions.

## Introduction

Mate preferences, and indeed mating behaviour, are strongly influenced by morphological traits exhibited by a potential partner [1], [2]. Potential mates must be assessed based on the costs and benefits associated with the traits they display. Both masculinity and symmetry are traits that can enhance physical attractiveness in humans [3], potentially because they are honest signals of male genetic quality [4]. Masculinity may signal immunocompetence and parasite load because testosterone, which triggers the development of masculine traits during adolescence, is an immunosuppressant and only individuals in good condition can afford to invest in secondary sexual traits [5], [6]. Symmetry may also indicate genetic quality. Bilateral symmetry is the developmental norm, and random deviations from symmetry (fluctuating asymmetries) are thought to reflect perturbations to normal development arising from a life-history of general poor health [for reviews see 7], [8]. Individuals of lower genetic quality are more likely to have disrupted developmental stability, and an associated increase in asymmetry. Therefore, a preference for masculine and/or symmetric traits could allow women to obtain mates in good condition, which might confer direct benefits such as lower contagion to the female, and/or, disease-resistance and other genetic benefits to her offspring.

There is evidence that women's mate preferences exhibit an adaptive shift that co-varies with the risk of conception [9], [10]. Several studies have shown an increase in women's visual preferences for relatively more masculine faces [e. g. 9], [10], [11], [12], bodies [13], [14], and voices [15], [16] during the fertile phase of the menstrual cycle. Furthermore, in a study of olfactory preferences, Havlicek, Roberts and Flegr [17] found a positive correlation between questionnaire-assessed male dominance, and women's perceptions of male odour ‘sexiness’ at their fertile point of the menstrual cycle, but not in other cycle phases. This cyclic effect on masculinity preferences may be influenced by relationship context. When both short- and long-term partner preferences were examined, a cyclic shift in visual preferences for masculinity were evident only when testing short-term partner preferences [10]. The same was true for olfactory preferences for psychological dominance [17].

An increased preference for masculinity at high fertility is argued to maximise potential genetic benefits that a masculine male might provide, while concurrently minimising associated costs. Masculinity has been associated with dominance and reduced parental care, and females must trade off genetic benefits against parental investment [e.g. 18]. This trade-off is supported by evidence that cyclic shifts are present only when selecting for a short-term, but not long-term partner [10]. A complementary hypothesis used to explain cyclic shifts in masculinity preferences argues for a central role for the hormone progesterone, the hormone that is present in elevated concentrations during pregnancy, and during the luteal phase of the menstrual cycle (after ovulation and before the onset of menses) [19], [20]. This argument proposes that a reduced preference for masculine men and an increased preference for relationship commitment during the luteal phase, reflects an adaptive strategy that may minimise costs and provide increased paternal investment during times when the hormone profile mimics pregnancy [19]. In summary, the benefits of a cyclic shift in preferences may be two-fold: maximising preferences for good genes to pass on to offspring (indirect benefits) when conception is likely, and increasing preferences for traits that signal parental investment (direct benefits) on days that reflect the hormone profile of pregnancy, and correspond with a low chance of conception.

A preference for symmetry in a potential mate might also maximise heritable fitness benefits. Although symmetry is generally attractive [see 3, for a review], evidence for an increase in the attractiveness of symmetry at high fertility is weaker than that for masculinity preferences. Several studies have shown an increase in female olfactory preference for symmetric males during high fertility days of the menstrual cycle (i.e., during ovulation) [21]–[24]. However, only one study has reported an increase in visual preferences for facial symmetry [25]. In contrast, three separate studies found no evidence of an enhanced visual preference for facial symmetry during the ovulatory phase [26]–[28]. There are no published studies that have investigated visual preferences for body symmetry over the menstrual cycle. Because body symmetry may be an indicator of general health [29], it may also be a good candidate for a preference that would be amplified at the fertile point.

With the exception of one study that investigated preferences for dominant behavioural displays in video recordings [30], prior menstrual cycle studies of visual preferences have not investigated cyclic shifts using naturally varying stimuli. In contrast with olfactory studies that are based on real odours of actual men (generally the stimuli are t-shirts worn by males for some time), studies of menstrual cycle shifts in visual preferences have used computer-generated faces that are manipulated to reflect different levels of symmetry or masculinity. All three studies of facial masculinity preferences used computer-generated composite stimuli, constructed by morphing several faces together, and then “masculinising” or “feminising” them [9], [10], [12]. This manipulation involves exaggerating or reducing features according to differences between an average male and average female configuration. For example as male jaws are larger than female jaws, a “masculinised” male face will have a larger jaw than the average male. Little et al.'s [13] study of masculinity in male bodies also used similar computer graphics techniques to construct masculinised and feminised versions of the same body. Likewise, the studies of cyclic shifts in visual preferences for symmetry also presented faces that were computer-altered to illustrate varying levels of symmetry [see 25], [26], [27], [28]. In all cases, female participants responded to different variations of the same male identity, either by choosing between a high and low trait version of the same face, or by selecting a preferred version of a face from a continuum. While this methodology may provide a very sensitive measure of women's perceptions, it is important to show that these preference shifts occur for unmanipulated faces and bodies to demonstrate the biological relevance of these findings.

No studies have shown any direct links between conception risk and preferences for morphological traits using photographs of real people. Attractiveness, symmetry and masculinity ratings of photographs of real male faces and bodies are significantly correlated with the photographed individual's mating success [2], [31]. Ratings of unmanipulated photographs therefore convey preferences that translate to actual female choice. It has been argued that female preferences for masculinity assessed using morphed faces also reflect actual female partner choice [32]. However, in that study, female participants who provided preference data also rated their own partner's masculinity, so ‘partner masculinity’ was not objectively assessed. Therefore, it may not be a reliable assumption that preferences during the menstrual cycle assessed using morphed faces will generalise to actual mate choice. An evolutionary-based argument that a cyclic shift in preferences reflects a female's increased attraction to traits that signal mate quality, would be strengthened if the effect was also evident when women rate photographs of real men, because of the applicability of ratings to actual mate choice.

The current study aimed to extend prior research by testing whether women's mate choice preferences change over the menstrual cycle when natural photographs of real men are used as stimuli. Women were tested at two sessions throughout their menstrual cycle: at ovulation (high-fertility) and during the luteal phase (low-fertility), and ovulation kits were used to determine the correct testing days. Short-term attractiveness ratings were targeted because previous research has shown a cyclic shift in short-term, but not long-term partner judgments [10]. These same male face and body photographs were also rated for masculinity and symmetry by two different groups of female raters, most of whom were using hormonal contraceptives which eliminate any cyclic effects [10], [33]. We correlated high- and low-fertile attractiveness ratings with these independent ratings of masculinity and symmetry and then compared preference strengths (i.e. correlations between attractiveness and masculinity, and attractiveness and symmetry) at the high- and low-fertile phase.

Using computer-generated stimuli, previous studies have found an increase in visual preferences at the high-fertility phase for masculinity, and have reported inconsistent results for symmetry. Our investigation of cyclic shifts in preferences for symmetry and masculinity in male faces and bodies is the first to a) use unmanipulated photographs of individual males, and b) to investigate preferences for body symmetry during the menstrual cycle. If evidence for a shift in symmetry or masculinity preference can be shown using photographs of real faces and bodies, this would provide strong evidence that a change in visual preferences throughout the menstrual cycle can be generalised to actual human mate choice.

## Results

**Figure 1:**
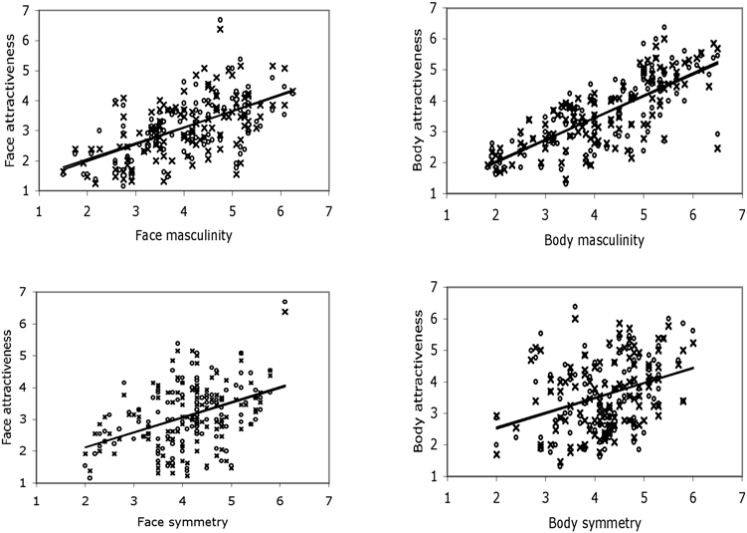
Scatterplots for high- and low-fertility ratings of attractiveness versus masculinity (top row) and symmetry (bottom row) for faces (left) and bodies (right). Lines of best fit are shown for both low- and high-fertility ratings. They overlap in each case so that only one line can be seen. × = low-fertility ratings. xˆ = high-fertility ratings.

**Figure 2:**
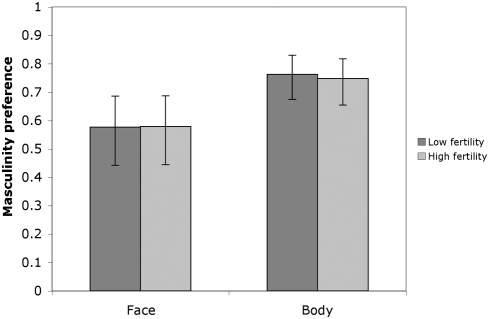
Masculinity preference strength as measured by Pearson correlation coefficients between attractiveness and each of face and body masculinity rated by women at low- (dark bars) and high- (light bars) fertility points of the menstrual cycle (n = 117, all *p* values<0.001). 95% CIs are shown.

**Figure 3:**
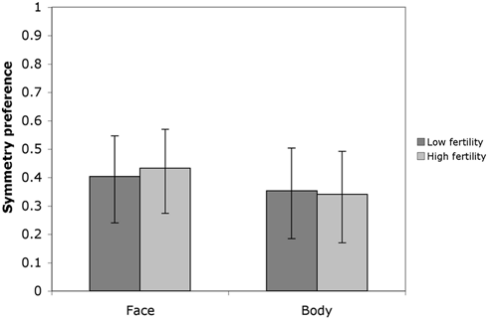
Symmetry preference strength as measured by Pearson correlation coefficients between attractiveness and each of face and body symmetry rated by women at low- (dark bars) and high- (light bars) fertility points of the menstrual cycle (n = 117, all *p* values<0.001). 95% CIs are shown.

Descriptive statistics for attractiveness, masculinity and symmetry ratings of each male face and body are shown in Table 1. Both face and body masculinity ratings were strongly and significantly correlated with low- and high-fertility attractiveness ratings (Figures 1 & 2). Similarly, face and body symmetry were also significantly correlated with attractiveness rated at both low- and high-fertility (Figures 1 & 3).

**Table 1:** Mean (±S.D) female ratings of male faces and bodies for all appearance variables.

	Male Faces (N = 117)	Male Bodies (N = 117)
Attractiveness: High-fertility	3.2 (1.0)	3.7 (1.1)
Attractiveness: Low-fertility	3.2 (1.0)	3.6 (1.1)
Masculinity	4.1 (1.0)	4.2 (1.2)
Symmetry	4.1 (0.9)	4.2 (0.8)

We formally compared high- and low-fertility preferences for masculinity and symmetry using Cohen and Cohen's [34] method for comparing dependent correlations with a shared variable. There was no significant difference between the high- and low-fertility preferences for masculinity in faces, *t*_114_ = 0.07, *p* = 0.94, or bodies, *t*_114_ = 0.44, *p* = 0.65. Nor was there any significant difference between high- and low-fertility preferences for symmetry in faces, *t*_114_ = 0.91, *p* = 0.37, or bodies, *t*_114_ = 0.45, *p* = 0.66.

There was no significant difference in high-fertility and low-fertility attractiveness ratings (averaged across male faces and bodies) for either face ratings, *t*_24_ = 0.20, *p* = 0.85 (high-fertility face rating: *M* = 3.3, *SE* = 0.1; low-fertility face rating: *M* = 3.3, *SE* = 0.1), or body ratings, *t*_24_ = 0.23, *p* = 0.82 (high-fertility body rating: *M* = 3.6, *SE* = 0.1; low-fertility body rating: *M* = 3.7, *SE* = 0.1).

## Discussion

This is the first study to measure women's visual preferences across the menstrual cycle using natural photographs of individual men. It is also the first to assess preferences for body symmetry across the cycle. We found no evidence for a cyclic change in preferences for either masculinity or symmetry in photographs of male faces or bodies. Furthermore, we found no significant difference between attractiveness ratings made at high- and low-fertility phases of the menstrual cycle. Our results are contrary to several studies which find an enhanced preference for masculinity when conception is likely for faces [9], [10], [12] and bodies [13]. These results also contrast with findings of an increased preference for facial symmetry during the fertile phase [25]. Instead they add to the body of literature that finds no evidence for a cyclic shift in visual preference for facial symmetry [see 26], [27], [28].

Given the numerous studies that have reported an increased preference for masculinity around ovulation, our results are perhaps surprising. It is unlikely that our testing method did not have the power to detect any changes in preferences. Our study was based on ratings of a relatively large number of stimuli (faces *N* = 117, bodies *N* = 117) compared with previous studies (e.g. [9] *N* = 5, [10] *N_study1_* = 10 & *N_study2_* = 5, [12] *N* = 1 continuum), and there was good variance within our ratings. While our female sample size (*N* = 25) was lower than that of previous studies (e.g. [9] *N* = 139, [10] *N_study1_* = 35 & *N_study2_* = 65, [12] *N* = 42), our Cronbach alphas showed very high agreement between raters (>0.95), suggesting that adding more female raters is unlikely to alter the outcome of our study.

We maximised the chances of detecting cyclic changes, if any were present, in several ways. First, we collected attractiveness ratings for a short-term partner. The menstrual cycle shift in masculinity preferences is stronger for short-term partner preferences [10], [12], [14]. Second, 80% of our female participants were in stable relationships at the time of testing, which may also maximise cyclic shifts in preferences [17].

Third, we defined the relevant days of the menstrual cycle as accurately as possible using ovulation predictor kits, in order to maximise the chances of detecting a cyclic shift in preference. Previously, most studies have relied on self-reported menstrual-cycle data and classified a wide range of cycle days as “fertile” [see for example 9], [10], [12]–[14], [17], [22], [25]–[27]. Self-reported cycle lengths are prone to measurement error, with 43% of women mis-reporting cycle length by 2 or more days in one study [35]. Additionally, using self-reported menstrual cycle data to estimate ovulation assumes that the luteal phase consistently begins 14 days prior to the onset of the next menses. However the luteal phase can vary between 4 and 19 days in length [36]. This self-reported menstrual cycle data therefore allows misclassification, so may underestimate any cyclic effects (although it does appear to provide accurate enough information to produce significant effects in some prior studies). Several studies have measured hormone levels around the testing days to confirm that ovulation had occurred during the relevant cycle [19], [25], [37]. While this method is useful for determining anovulatory cycles, it is less useful for measuring the precise day of ovulation. Only two menstrual cycle studies have confirmed the occurrence and the date of ovulation during the high-fertile testing phase, but they were studies of female sexual interest across the cycle [38], [39], not of visual preferences. Ovulation predictor kits are >99% accurate in detecting a pre-ovulatory lutenising hormone surge [40, Mentholatum Australasia Pty. Ltd, Key Pharmaceuticals], and 97% accurate in predicting ovulation when confirmed using ultrasound [40], The present study employed ovulation predictor kits to ensure that women were correctly classified at high fertility, and also to allow the exclusion of anovulatory cycles.

Fourth, classification of female participants as low and high fertility at the luteal phase (rather than at menses or before ovulation) and at ovulation respectively, maximises differences not only between fertility levels, but also between progesterone levels during the menstrual cycle [19], [and see 20], [41]. Testing at these two phases of the cycle may also capture preference differences attributable to testosterone levels [see 37]. Although not maximally different, there is a modest decrease in total testosterone levels from ovulation to the luteal phase [42].

Taken together, short-term partner attractiveness ratings and the relationship status of the majority of the women, combined with the use of ovulation predictor kits and low- and high fertility classification should have increased the likelihood of finding cyclic shifts in female mate preferences, and still none were evident.

Although findings from prior studies of visual preferences for masculinity during the menstrual cycle may be robust, our study suggests that the menstrual cycle effect may be contingent on the use of computer-generated faces or bodies. Each study that found an enhanced visual preference for masculinity during ovulation, presented experimental stimuli that were manipulated to vary only in masculinity levels [9], [10], [12], [13]. The artificial stimuli employed might enhance the detection of a preference shift above what might be evident in natural populations, because of the uni-dimensional face variation. Interestingly, however, Gangestad et al. [30] found a cyclic shift in preferences for dominant behaviours in video clips of males. While that study did not assess face or body masculinity *per se*, it seems to be in contrast to our findings using photographic stimuli and warrants further investigation of masculinity in video clips and dominance in photographs to help elucidate the generalisation of these findings to other media. Our study also differs from many (but not all) other studies of cyclic preferences in its use of ratings of individual faces rather than forced-choice preferences between pairs of faces that differ on a single dimension [see for example 9], [10], [13], [14], [19], [20]. Although the forced choice method may be more sensitive to cyclic changes than a ratings design, rating an image, relative to a large number of other images, is arguably more relevant to the mate choice decision-making process, particularly when the forced choice is made between two versions of the same face or body.

There is some evidence that symmetry detection may improve around ovulation, compared with the luteal phase [28], but a similar increase in symmetry preferences is less clear. Although one study has found an increase in facial symmetry preferences around ovulation using the computer-generated stimuli method [25], three other studies that used similar methodologies found no evidence for a cyclic shift in symmetry preferences [26]–[28]. Our study using natural images adds to the converging evidence against cyclic shifts in symmetry preferences, and suggests that even when a shift is found, the change in preference may not be relevant to mate choice decisions. Although our results, as well those from other studies, suggest that there is no cyclic shift in visual preferences for symmetry, there is, nevertheless, evidence for an increase in preference for the smell of symmetric men at the fertile point of the menstrual cycle [21]–[24]. This may be because women develop a more sensitive sense of smell during ovulation [43], which may allow them to assess subtle cues which may not be evident during other times of the cycle.

In this study we analysed women's perceptions of symmetry and masculinity using ratings. Although symmetry and masculinity ratings correlate significantly with measured fluctuating asymmetry (FA) and masculinity respectively, we cannot be certain that the same results would be obtained with measurements of these traits. However, female mate choice decisions are made in response to perceived symmetry and masculinity, so ratings that reflect these perceptions are a biologically valid proxy for use in preference studies. Further research, however, might use measurements from real (not computer generated) faces and bodies to examine preferences for measured symmetry and masculinity throughout the menstrual cycle.

Penton-Voak et al. [10] argue that sexual behaviour that arises from cyclic female preferences may allow a female to gain heritable benefits from a masculine male, whilst maintaining a less masculine partner when conception is less likely may confer parental care benefits. Our results raise doubts over whether changes in visual preferences during the menstrual cycle actually affect mate choice and sexual behaviour. For this set of male faces attractiveness ratings are correlated with actual mating success [31], but it appears that masculine males are no more attractive, and therefore no more likely to be chosen as a mate, when females are ovulating.

A consistent, strong preference for masculinity throughout the menstrual cycle may reflect a preference for a healthy mate [44] which would confer benefits to the female whether or not she is at the fertile point of her cycle. Similarly, a consistent preference for symmetry in a potential mate throughout the menstrual cycle may also be adaptive. Higher levels of symmetry are thought to result from an ability to withstand exposure to pathogens and toxins, because of “good genes” [45]. A partner with good genes may therefore provide indirect benefits via the production of superior offspring, and direct benefits if good genes are associated with lower contagion, which would be advantageous to a female at any point of the cycle.

In summary, although there is strong evidence for an increase in preference for masculinity when the chance of conception is high in studies using artificial stimuli, the present results suggest that a menstrual cycle shift may not affect preferences for real faces and bodies. We also found no evidence for a shift in symmetry preferences, which replicates most previous studies of visual preferences for facial symmetry and extends the findings to body symmetry. Preferences for masculinity and symmetry were equally strong at both high- and low-fertility phases of the menstrual cycle. These results suggest that the subtle menstrual cycle shifts found in studies using computer-generated stimuli may not influence actual female mate choice. Additional research into masculinity and symmetry preferences using more realistic mate choice scenarios and stimuli (e.g. video recordings) may further help to determine whether cyclic shifts in preferences are applicable to biologically relevant situations. Our results suggest that caution should be exercised over the extent to which findings for preference shifts over the menstrual cycle are generalised to actual mate choice.

## Methods

### Raters

Twenty-seven Caucasian females with regular menstrual cycles participated after giving written, informed consent. Participants were not currently using any form of hormonal contraception. Two of these females did not ovulate after testing during three cycles and were excluded from analyses. The mean age of the remaining 25 participants was 28.9 years (S.D. = 3.4, range = 23–34 years) and 80% of these women were in a stable relationship at the time of testing. Females in this sample had a mean cycle length of 29 days (S.D. = 1.9, range = 25–32 days).

A further 24 women also participated in the study to provide independent ratings of masculinity and symmetry. Most of these raters (88%) were taking hormonal contraception and the ratings were not obtained on particular days of their menstrual cycle. The mean age of these 24 women was 21.6 years (S.D. = 4.9, range = 17–34 years). These women are a subset of the raters described in Peters et al. [31]

### Stimuli

**Figure 4:**
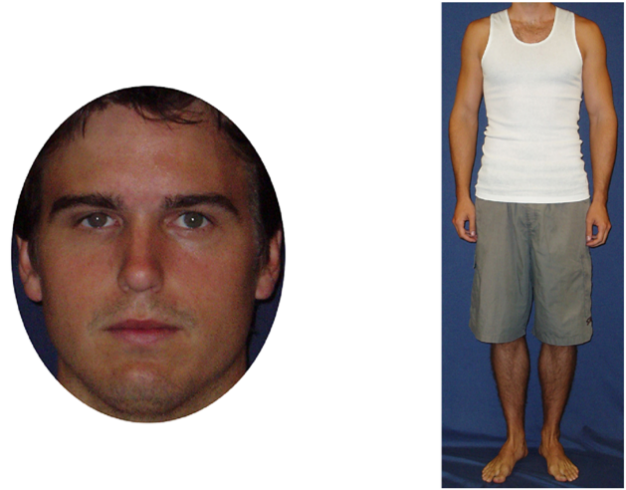
Example of male face and body photographs presented for ratings.

Front-view face and body colour photographs of 117 adult caucasian males were used as stimuli for the attractiveness ratings [46] (Figure 4). These stimuli have been used and described in two previous studies [31], [46]. In the body photographs, the men wore white fitted singlets and shorts, and stood with arms relaxed by their sides and heels aligned. Face photographs displayed a neutral facial expression, and were cropped so that most hair cues were removed. Photographs were presented on a computer screen at 72 pixels per inch, with a constant height (320 pixels for faces and 480 pixels for bodies) and varying width.

### Procedure

Participants rated the face and body photographs for attractiveness at high- and low-fertile points of the menstrual cycle. Each participant used an *Ovuplan* (Key Pharmaceuticals) or *Confirm* (Mentholatum) ovulation predictor kit that determines a surge in luteinizing hormone (LH): the hormone that triggers ovulation. Within 48 hours of the LH surge, females rated the faces and bodies for attractiveness. Women were therefore tested on the day before, or the day of ovulation, when the probability of conception is highest [47]. This was termed the “high-fertility” testing session. Four participants did not detect a LH surge during their first monitored cycle, so were given another ovulation kit to test again during the next cycle, and ovulation was subsequently confirmed.

Females also rated the faces and bodies for attractiveness during the luteal phase of the menstrual cycle. This phase occurs after ovulation but before the onset of menses, and is associated with a very low chance of pregnancy (henceforth termed the “low-fertility” testing session). Order of testing was balanced across participants (half first rated the images at ovulation and second during the luteal phase, and half vice versa).

The faces and bodies were rated for attractiveness on a 7-point scale (1 = not attractive at all, 7 = very attractive). Females were asked to rate in terms of sexual attractiveness, as if they were rating a potential short-term partner, and were encouraged to use the full range of the rating scale. The photographs were blocked by image type (face or body) and presented randomly within each block. Order of image type presentation was also balanced, with half of the participants in each testing order (ovulation-luteal or luteal-ovulation) rating faces first and bodies second, and half vice versa. Images remained on the screen until a rating was made. There was very high agreement on attractiveness ratings between raters with all Cronbach alphas >0.95 (*M* = 0.95, *S.D.* = 0.01).

Independent ratings of masculinity and symmetry were also collected using 7-point scales. Twelve women rated masculinity and 12 different women rated symmetry in the face and body photographs. These women rated the photographs only once, using the same method of image presentation as outlined above. Because most of the masculinity and symmetry raters were taking hormonal contraceptives, no cyclic effects were expected in these rater groups [10], [33]. As for the attractiveness ratings, mean Cronbach alphas for face and body masculinity and symmetry were high (masculinity *M* = 0.91, *S.D.* = 0.04, symmetry *M* = 0.73, *S.D.* = 0.06).

Importantly, symmetry ratings have been shown to predict measured symmetry in faces [48]. More specifically, symmetry ratings are associated with measured fluctuating asymmetry, which is proposed to reflect developmental instability and be an honest indicator of mate quality [48]. Furthermore, rated masculinity is significantly correlated with measurements of sexually dimorphic features in male faces [49]. Therefore human perceptions of symmetry and masculinity (as measured by ratings) can be argued to be true reflections of actual trait levels.

### Statistical analyses

First, ratings were averaged across female raters to provide high- and low-fertility ratings of attractiveness for each individual male face and body. Correlations between masculinity and attractiveness at high-fertility were compared with those at low-fertility, and the same was done for ratings of symmetry.

Second, low-fertility face, low-fertility body, high-fertility face, and high-fertility body attractiveness ratings were calculated for each female rater by averaging across all male faces and all male bodies. We then performed a within-subject comparison of the high- and low-fertility ratings to assess whether attractiveness ratings changed across the menstrual cycle.
